# Investigation of the Mechanical and Dynamic-Mechanical Properties of Electrospun Polyvinylpyrrolidone Membranes: A Design of Experiment Approach

**DOI:** 10.3390/polym12071524

**Published:** 2020-07-09

**Authors:** Andrea Dodero, Elisabetta Brunengo, Maila Castellano, Silvia Vicini

**Affiliations:** 1 Department of Chemistry and Industrial Chemistry (DCCI), University of Genoa, Via Dodecaneso 31, 16146 Genova, Italy; elisabetta.brunengo@scitec.cnr.it (E.B.); silvia.vicini@unige.it (S.V.); 2 Institute of Chemical Sciences and Technologies “Giulio Natta” (SCITEC), Italian National Research Council (CNR), Via De Marini 6, 16149 Genova, Italy

**Keywords:** electrospinning, polyvinylpyrrolidone, aligned nanofibers, mechanical properties, dynamic mechanical analysis, design of experiment

## Abstract

Polyvinylpyrrolidone electrospun membranes characterized by randomly, partially, or almost completely oriented nanofibers are prepared using a drum collector in static (i.e., 0 rpm) or rotating (i.e., 250 rpm or 500 rpm) configuration. Besides a progressive alignment alongside the tangential speed direction, the nanofibers show a dimension increasing with the collector rotating speed in the range 410–570 nm. A novel design of experiment approach based on a face-centred central composite design is employed to describe membrane mechanical properties using the computation of mathematical models and their visualization via response surface methodology. The results demonstrate the anisotropic nature of the fibre-oriented membranes with Young’s modulus values of 165 MPa and 71 MPa parallelly and perpendicularly to the alignment direction, respectively. Above all, the proposed approach is proved to be a promising tool from an industrial point of view to prepare electrospun membranes with a tailored mechanical response by simply controlling the collector speed.

## 1. Introduction

The electrospinning technique has emerged in the last decade as a powerful and relatively affordable approach to prepare membranes composed of fibres with diameters ranging from a few nanometres to several microns [1,2]. The basic principle behind electrospinning is the application of a high voltage to a polymer solution or melt to generate repulsive forces between the macromolecules. When such forces overcome the system surface tension, a continuous polymer jet is ejected, usually from the tip of a needle, and collected on a proper surface, taking advantage of the complete solvent evaporation occurring during the flight time [3,4]. Due to the great versatility and the high production rate compared to other fibre-forming technique [5], electrospinning has gained increasing interest from both the academic and industrial points of view, with a great range of possible applications and, indeed, several patents have been already registered [6,7,8]. For example, several electrospun membranes based on both synthetic and natural polymers were prepared for biomedical [9,10,11,12,13,14,15], environmental [16,17,18], air filtration [19,20,21], energy storage [22,23], sensing [24,25], and textile purposes [26,27], thus showing the huge future potentialities of this technique. The typical electrospinning set-up leads to randomly oriented nano- or microfibers exhibiting isotropic mechanical properties which usually show a lesser performance compared to other types of polymeric products (e.g., films, hydrogels, etc.) [28,29]. To overcome such limitations, the use of engineered collectors able to promote the fibre orientation along single or multiple directions has been recently and successfully investigated, giving rise to the development of extremely diversified structures with peculiar properties [30,31,32,33]. Besides the fact that most of these set-ups are not easily scaled-up and remain a niche production, rotating drum collectors represent a promising approach at an industrial level to prepare membranes with oriented nanofibers showing improved and anisotropic mechanical properties that can be easily tuned by controlling the processing parameters [34,35]. Therefore, a deep understanding of the several experimental variables affecting the sample properties it is extremely important [36,37]; however, modelling the mechanical response of so-prepared products still represents a great challenge, and systematic investigations are scarcely reported. In this regard, the design of the experiment is considered an encouraging approach to achieve this kind of knowledge, as it is able to evaluate simultaneously the influence of several variables and, above all, the existence of synergic or opposite interactions between them. In particular, by investigating the system response in well-defined points of the experimental domain, it is then possible to compute the obtained data to derive a mathematical model able to describe the property of interest within all of the variable ranges [38,39,40,41,42,43]. 

In the present work, polyvinylpyrrolidone electrospun membranes were prepared using a drum collector rotating at different speeds to obtain structures with randomly, partially, and nearly completely organized nanofibers. The morphology of the prepared samples was evaluated via scanning electron microscopy and the diameter distribution of the fibres investigated. A complete screening of the sample mechanical properties was then performed according to a face-centred central composite design; in particular, uniaxial tensile tests, dynamic-mechanical, and dynamic-mechanical-thermal analysis were performed on the prepared samples parallelly, transversely, and perpendicularly to the fibre alignment direction. Finally, the Young’s modulus, the maximum tensile strength, and the elongation at break data were elaborated to derive simple mathematical models able to fully describe the sample anisotropic response with a surface response methodology used to rapidly visualize the effects of the investigated variables. To the best of our knowledge, this is the first time that such an approach was employed to evaluate the properties of electrospun membranes and efficaciously proved to be a very important tool to precisely obtain products with a tuned mechanical response.

## 2. Materials and Methods 

### 2.1. Materials

Polyvinylpyrrolidone (PVP) with an average number of molecular mass ${\bar{M}}_{n}$ = 750 kg/mol and absolute ethanol (EtOH) were purchased from Sigma Aldrich.

### 2.2. Methods

#### 2.2.1. Solution Preparation and Electrospinning

PVP solutions with a concentration of 10 % *w*/*v* were prepared by dissolving the polymer powder in absolute ethanol for 24h under slow stirring to ensure complete solubilization and homogeneity. 

A Doxa Microfluidics Professional Electrospinning Machine was used to prepare the nanofibrous membranes. A metallic drum collector with a diameter of 20 cm was employed to collect the fibres with rotating speeds of 0 rpm, 250 rpm, or 500 rpm. In a typical experiment, 20 mL of PVP solution was electrospun using a spinneret-collector distance of 15 cm, an applied voltage of 12 kV, an infuse rate of 1 mL/h, and a needle with an inner diameter of 0.4 mm. 

#### 2.2.2. Scanning Electron Microscopy (SEM)

The morphology of the electrospun membranes was evaluated using a Hitachi TM3000 benchtop scanning electron microscope (Hitachi High-Tech GLOBAL, Tokyo, Japan) operating at an acceleration voltage of 5 kV. Prior to observation, the surface of the samples was sputtered with silver using a Quorum Q150R ES sputter coater (2 M Strumenti, Rome, Italy) at 50 mA for 2 min.

SEM images were analysed with the open-source Image J 1.51 software (National Institute of Health, Bethesda, MD, USA) to evaluate the fiber average size, and at least 200 measurements were taken for each sample.

#### 2.2.3. Design of Experiments

A design of experiments (DoE) approach was applied in order to derive a mathematical model able to predict the mechanical response of the prepared membranes depending on the direction of the investigation and on the applied collector rotating speed, corresponding to a different fibre alignment. A face-centred central composite design (FCCD) was employed to determine the linear and the quadratic terms as well as the interactions occurring between the investigated variables according to Equation (1):

(1) $$y=b_{0}+b_{1}x_{1}+b_{2}x_{2}+b_{12}x_{1}x_{2}+b_{11}x_{1}^{2}+b_{22}x_{2}^{2}$$

where y is the system response, b_0_ is a constant term, x_1_ and x_2_ are the independent variables (i.e., collector rotating speed and direction of the applied stress), b_1_ and b_2_ are the linear coefficients, b_11_ and b_22_ are the quadratic coefficients, and b_12_ is the interaction coefficient. Such a model is based on a number of experiments n equal to:

(2) $$n=2^{k}+2k+m$$

where k is the number of variables and m indicates the number of replicates in the central point of the experimental domain. In the present work, given k = 2 and m = 2, a total number of 10 experiments was performed. Table 1 summarizes the experimental variables and their investigated ranges.

The Young’s modulus (Y), the maximum tensile strength (σ_r_), and the elongation at break (ε_r_) were modelled according to the presented approach and, finally, a response surface methodology (RSM) was employed to rapidly visualize the effect of the selected variables on the membrane mechanical properties.

All data were elaborated using the free source Chemometric Agile Tool (CAT) software.

#### 2.2.4. Mechanical and Dynamic-Mechanical Characterization

The mechanical behaviour of the electrospun membranes prepared with a different collector rotating speed was first evaluated using uniaxial tensile tests performed by means of a displacement-controlled dynamometer Instron 5565 (Instron, Turin, Italy). Rectangular specimens (40 mm × 10 mm × 0.15 mm) were cut from the membranes using a punch cutter alongside (i.e., with an angle of 0°), transversely (i.e., with an angle of 45°), and perpendicularly (i.e., with an angle of 90°) to the tangential velocity direction. The samples were fixed on smooth pressure clamps applying a pre-load of 0.1 N to ensure the correct sample loading; the measurements were carried out at room temperature with an elongation rate of 25 mm/min, and each experiment was repeated five times to ensure the repeatability of the results. The mechanical properties (i.e., Y, σ_r_ and ε_r_) of the samples were then derived from the obtained stress-deformation plot, with the stress (MPa) and the deformation (%) calculated according to Equations (3) and (4), respectively:

(3) $$\sigma =\frac{F}{A}$$

(4) $$\varepsilon =\frac{l-l_{0}}{l_{0}}\cdot 100$$

where F represents the applied load, A the section of the sample, l_0_ the initial sample length, and l the sample length at a certain time.

The dynamic-mechanical properties of the electrospun membranes were assessed using a rotational rheometer Anton Paar MCR 301 (Anton Paar GmbH, Graz, Austria) equipped with a universal extensional fixture (UXF) geometry and a CDT-450 chamber. Rectangular specimens (40 mm × 10 mm × 0.15 mm) were cut from the membranes using a punch cutter alongside (i.e., with an angle of 0°) and perpendicularly (i.e., with an angle of 90°) to the tangential velocity direction. In all cases, a pre-stress of 1 MPa was applied to ensure the correct sample loading and reliability of the obtained results; note here that in dynamic mechanical tests the applied pre-stress must be mandatorily higher than the oscillating stress. Firstly, amplitude sweeps were performed at T = −40 °C, T = 20 °C, and T = 80 °C to determine the linear viscoelastic range (LVER) of each sample using a fixed frequency ν of 1 Hz and oscillating stress σ ranging from 0.05 MPa up to 0.5 MPa. Secondly, frequency sweeps were carried out in a frequency range of 0.1–10 Hz by applying oscillating stress of 0.1 MPa and varying the temperature from −40 °C to 80 °C with ΔT = 30 °C. Finally, temperature sweeps were conducted in the temperature range −40–80 °C with a heating rate of 2 °C/min and a frequency of 1 Hz by applying oscillating stress of 0.1 MPa. 

## 3. Results

### 3.1. Morphology

One of the main advantages of the electrospinning technique is the possibility to obtain membranes constituted by nano and/or microfibers whose dimensions and spatial distributions can be easily controlled by changing the solution (e.g., polymer concentration and used solvent) and the processing (e.g., applied voltage, spinneret-collector distance, infuse rate, needle diameter, type of collector, etc.) parameters, therefore making it possible to obtain a wide range of structures with different properties [44,45,46].

**Figure 1:**
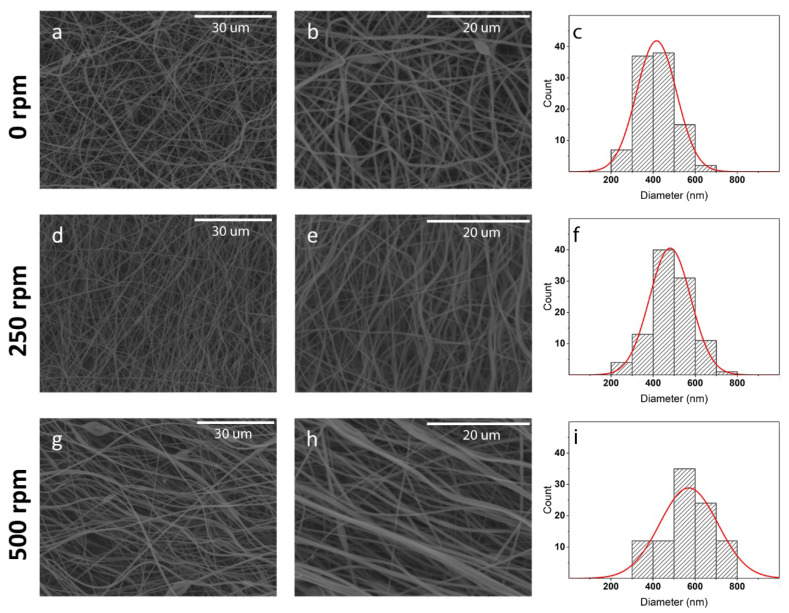
The morphology at low (**a**, **d** and **g**) and high (**b**, **e**, and **h**) magnification for PVP membranes obtained with a collector rotation speed of 0 rpm (**a** and **b**), 250 rpm (**d** and **e**), and 500 rpm (**g** and **h**). Size histograms (**c**, **f** and **i**) of the obtained nanofibers are given.

Figure 1 shows the morphology of the prepared PVP membranes obtained using a cylindrical drum collector rotating at different speeds, from randomly (i.e., rotating speed of 0 rpm) to oriented (i.e., rotating speed of 500 rpm) nanofibers.

**Figure 2:**
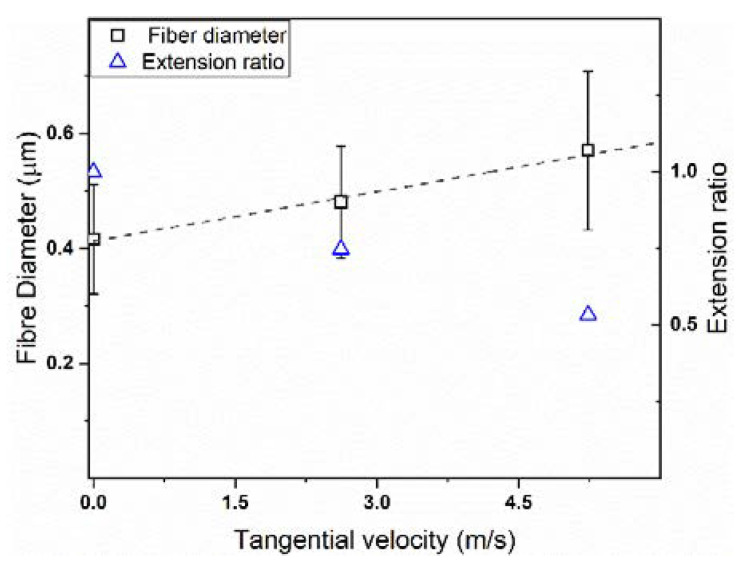
Fibre diameter (square symbols) and extension ratio (triangular symbols) dependence upon the collector velocity.

Smooth and almost defect-free fibres were obtained independently on the collector rotating speed, which was not surprisingly owing to the high spinnability of PVP in pure ethanol [47,48]. However, significant differences can be observed for the three prepared membranes both in terms of fibre dimension and orientation. Randomly-oriented nanofibers with a diameter of 410 ± 100 nm (Figure 1a,b) were obtained when the drum collector was stationary; on the contrary, partially-oriented nanofibers with a diameter of 480 ± 100 nm (Figure 1d,e) and considerably oriented nanofibers with a diameter of 570 ± 140 nm (Figure 1g,h) were obtained with a collector rotation speed of 250 and 500 rpm, respectively [49,50]. Figure 2 reports the fibre diameter dependence upon the collector tangential speed.

Two distinct effects can be observed regarding the dependence of the collector rotation speed. Firstly, the orientation of the fibres along a certain direction was amplified as the collector rotated at a higher speed in agreement with the literature [51,52]. Such a phenomenon is due to the fact that higher rotation speeds correspond to a greater tangential velocity of the drum surface, and consequently, the nanofibers are preferentially packed following that same direction. Intuitively, the polymer jet is subjected to greater elongational forces as the collector rotates more rapidly, and thus thinner nanofibers should be obtained; however, contrariwise to what is expected, an increase of the fibre dimension and a wider diameter distribution was observed here, increasing the collector rotating speed. To better understand this unexpected finding, the extension ratio λ was calculated according to Equation (5) [35]:

(5) $$\lambda =\frac{d_{1}^{2}}{d_{2}^{2}}$$

where d_1_ and d_2_ are the diameter of the fibres obtained with the static and the rotating collector, respectively. The results are shown in Figure 2.

As observed, the fibre diameter linearly increased with the collector tangential speed, whereas the opposite trend was followed by the extension ratio. Such a finding is most likely related to two different causes. Firstly, at higher rotating speeds, the electrical driving forces to which the polymer jet is subjected are amplified by the mechanical stretching induced by the collector; consequently, the flight time of the created jet is strongly reduced, preventing the complete evaporation of the solvent and leading to greater fibre dimension. Secondly, as suggested by the wavy shape assumed by the aligned fibres and clearly visible in Figure 2g, once the membranes are removed from the collector, they probably undergo to an elastic retraction that, in turn, leads to an increase of the fibre diameter. Indeed, besides the macroscopic orientation of the nanofibers, the use of a high rotating speed is also able to induce the microscopic orientation of the polymer chains alongside the fibre direction, which, however, tend to partially return to a more thermodynamically-favourable conformation once the stretching forces are removed [53,54,55,56,57,58].

### 3.2. Mechanical Properties

Compared to the correspondent polymer films, electrospun membranes are usually characterized by poorer and isotropic mechanical properties in terms of Young’s modulus and maximum tensile strength [34]. However, when the nanofibers are partially or completely aligned, the membranes can assume anisotropic properties, and a strong rise of the mechanical behaviour can be obtained in a certain direction. 

**Figure 3:**
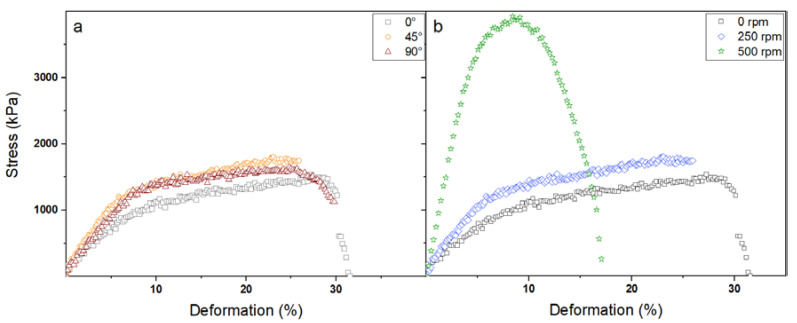
Stress-deformation curves for PVP membranes obtained with the same collector rotating speeds (i.e., 0 rpm) in different directions (**a**) and for PVP membranes obtained with different collector rotating speed in the same direction (i.e., 0°) (**b**).

Figure 3a shows the stress-deformation curve for the sample obtained with the static collector (i.e., 0 rpm) in three different directions with respect to the fibre alignment (i.e., 0°, 45° and 90°). Figure 3b reports the stress-deformation curve for the samples obtained with different rotating speeds (i.e., 0 rpm, 250 rpm, and 500 rpm) tested along the tangential speed direction. As expected, no differences can be detected in terms of mechanical properties for the samples obtained with the static collector independently on the investigated direction (Figure 3a); indeed, since the nanofibers are randomly oriented within the membrane structure, isotropic behaviour is clearly observed. However, aligned nanofibers led to a significant rising of the sample mechanical properties alongside the direction of orientation (Figure 3b); in particular, the fibre alignment corresponds to a higher Young’s modulus, a higher maximum tensile strength, and a lower elongation at break, with the sample obtained at rotating speed of 500 rpm showing the greatest mechanical performances. Besides the fibre alignment itself, the electrostatic and, above all, the mechanical stretching forces taking place during the electrospinning process are able to induce a marked orientation of the polymer chains within the nanofibers, which in turn contributes to strongly increase the mechanical stiffness of the membranes, leading to higher Y and σ_r_ values but to a lower deformation capability.

Table 2 summarizes the Young’s modulus, the maximum tensile strength, and the elongation at break values of all the tested membranes.

By analysing the obtained data, it can be observed that a higher alignment alongside the fibre direction corresponds to greater values of Y (increase of 61% and 275% for the samples obtained with a rotating speed of 250 rpm and 500 rpm, respectively) and σ_r_ (increase of 17% and 230% for the samples obtained with a rotating speed of 250 rpm and 500 rpm, respectively) but lower values of ε_r_ (decrease of 27% and 63% for the samples obtained with a rotating speed of 250 rpm and 500 rpm, respectively). Surprisingly, also at 45° and 90° with respect to the collection direction, the membranes with aligned nanofibers were characterized by higher Young’s modulus values compared to the one obtained with the static collector, owing to the orientation of the polymer chains themselves that provides further mechanical reinforcement; nevertheless, as expected, a marked decrement of Y can still be observed transversely and perpendicularly compared to the alignment direction due to the lower ability of the nanofibers to sustain a certain stress. Regarding the maximum tensile strength, besides the fact that membranes with aligned fibres showed a significantly greater resistance alongside the orientation direction compared to the randomly-organized one, lower (or similar) σ_r_ values were observed at 45° and 90° with respect to the fibre direction, especially for the sample obtained with a rotating speed of 250 rpm. This is most likely because, when the polymer chains are partially orientated in a certain direction, their ability to respond to mechanical stress applied in a different direction is somehow negatively affected. Finally, for the aligned-organized membranes, a significant increase of the elongation at break was observed at 45° and 90° with respect to the fibre orientation direction. Such a result is probably related to two different processes occurring during the sample mechanical deformation. On the one hand, the stretching of the membranes induces a progressive dealignment of the nanofibers, which in turns provides a greater elongation capability before the rupture; similarly, the polymer chains are somehow forced to assume a more disordered conformation, thus providing higher deformability to the membranes transversely and perpendicularly to the initial alignment direction.

### 3.3. Dynamic-Mechanical Properties

**Figure 4:**
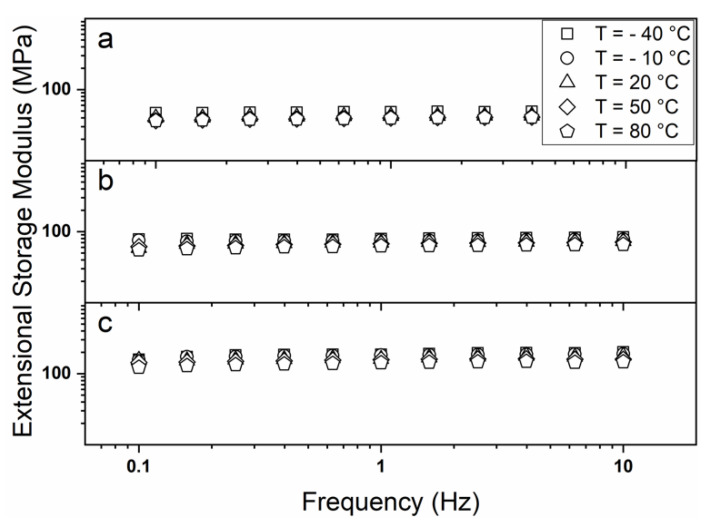
Dynamic-mechanical behaviour in the temperature range −40 to 80 °C for PVP membranes obtained with a collector rotation speed of 0 rpm (**a**), 250 rpm (**b**), and 500 rpm (**c**).

Besides the commonly employed uniaxial tensile test, dynamic-mechanical (DMA) and dynamic-mechanical-thermal (DMTA) analysis can provide a wide range of information regarding the reaction of a polymeric material subjected to different types of stress. Firstly, DMA was used to evaluate the mechanical response of the prepared membranes when oscillating constant stress was applied at different a temperature and with a different frequency. Figure 4 shows the obtained results for the prepared membranes alongside the nanofiber collection direction (i.e., 0°), with a similar behaviour observed perpendicularly (i.e., 90°). 

In the investigated frequency range, the samples showed a nearly constant trend of the extensional storage modulus E’, as well as a slight decrease of the extensional loss modulus E” (data not shown), which is consistent with their predominantly elastic nature and localizes their response within the rubbery elastic plateau region [59,60,61]. The DMA spectrum of polymers can be indeed divided into several portions depending on the material properties. At low frequencies, which cannot usually be experimentally investigated for solid samples, the terminal or viscous regime is observed with both E’ and E” increasing. At intermediate frequencies, the rubbery elastic plateau region is reached with the material showing a constant value of E’ and a more or less marked decrement of E”, indicating the tendency to store rather than dissipate energy. Increasing the frequency, firstly a transition region in which the viscous and the elastic response of the material are somehow comparable occurs, and finally the glassy state occurs, where for both E’ and E” a constant value (E’ >> E”) is reached [62,63]. Moreover, a decrease of the extensional elastic modulus was observed by raising the investigation temperature, owing to the higher mobility of the polymer chains; besides, no significant changes in the mechanical response could be detected, thus indicating the good thermal stability of the prepared PVP electrospun membranes. In Table 3, a summary of the membrane dynamic-mechanical response is reported. Note that a strikingly good agreement between the Young’s modulus E (Table 2) and the extensional elastic modulus E’ was achieved.

**Figure 5:**
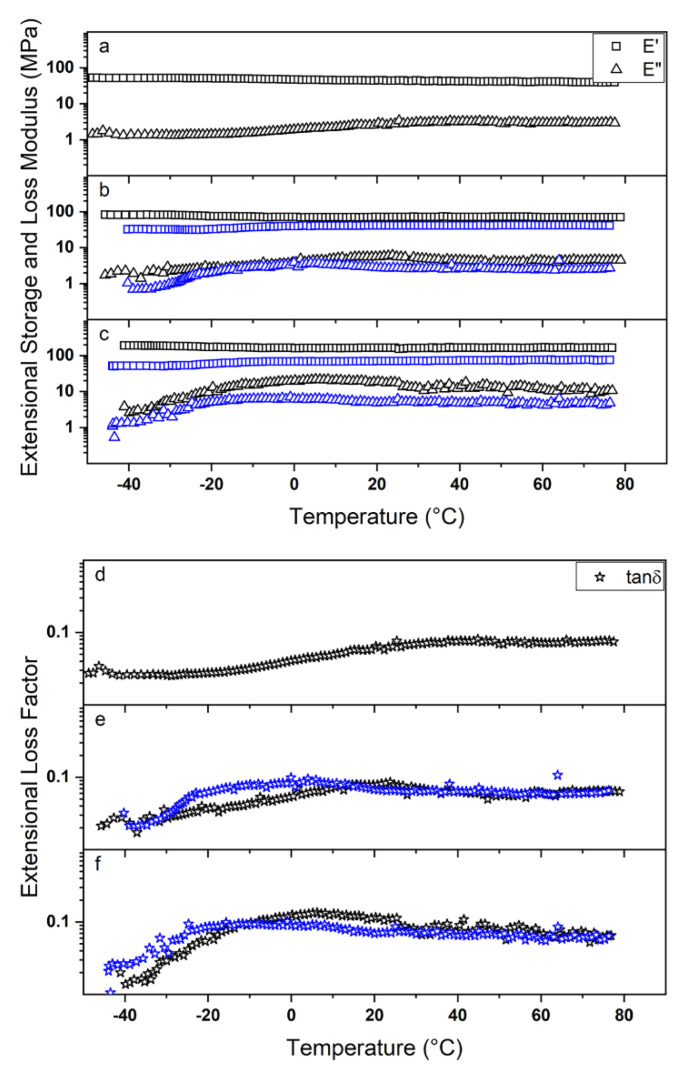
Dynamic-mechanical-thermal behaviour for PVP membranes obtained with collector rotation speeds of 0 rpm (**a**–**d**), 250 rpm (**b**–**e**), and 500 rpm (**c**–**f**). Black and blue symbols represent the properties along and perpendicularly to the fibre orientation direction, respectively. Square, triangle, and star symbols indicate the Extensional Storage Modulus, the Extensional Loss Modulus, and the Extensional Loss Factor, respectively.

The dynamic-mechanical-thermal properties of the prepared membranes were also investigated to better understand the moduli temperature dependence and in order to detect the eventual presence of secondary transition phenomena [64,65,66,67,68]. Figure 5a–c show the moduli mechanical spectra for the prepared membranes.

All samples, independently on both the collector rotating speed and the direction of the investigation, showed a good consistency with the results obtained from the uniaxial tensile tests and the dynamic-mechanical analysis. However, important differences can be observed depending on the investigated direction with respect to the fibre alignment; indeed, besides E′ predominated E″ in the whole temperature range, a different trend of the moduli was observed. Alongside the fibre direction (black symbols in Figure 5a–c) a more or less constant decrement of E’ was detected as the temperature was raised, whereas E” showed a trend at maximum before assuming a constant value; indeed, as the thermal energy increases, the material abilities to store and dissipate energy are negatively and positively affected, respectively. By evaluating the extensional loss factor ($\tan\delta =\frac{E\prime \prime }{E\prime }$) spectra alongside the collecting direction (i.e., 0°) and reported in Figure 5d–f (black symbols), a trend at maximum can be again observed. These tanδ peaks correspond to a secondary transition phenomenon that is most likely related to an entropic retraction of the PVP chains within the nanofibers; in particular, the more aligned the polymer chains are (corresponding, as explained above, to more aligned fibres due to the stretching forces occurring during electrospinning), the lower the temperature T_φ_ at which such retraction is detected. Perpendicularly to the orientation of the fibres (blue symbols in Figure 5b,c) a slight increase of E’ can be observed at around T = −20 °C; such a finding is most likely ascribable to a progressive macroscopic orientation of the nanofibers along the direction of mechanical solicitation, which in turn leads to an improved membrane elastic response. Taking into account the abovementioned secondary transition perpendicular to the fibre alignment direction (blue symbols in Figure 5e,f), a decrement of the occurring temperature T_φ_ can be observed compared to the direction of orientation. The mechanical solicitations to which the nanofibers, as well as the polymer chains, are orthogonally subjected, play indeed a synergic role with the thermal energy, promoting the entropic retraction phenomenon which occurs at a lower temperature. On the contrary, when the mechanical solicitations occur along the fibre axis, they do not influence the process. The transition temperatures T_φ_ are summarized in Table 3. 

### 3.4. Model Computation

The design of experiments is a relatively novel approach that allows us to obtain mathematical models able to describe the response of a real system by simply computing the results obtained in selected points of the experimental domain. Besides the possibility to predict a priori the system response, such a method can be used to obtain important information about the influence of the investigated variables and their interactions, thus pointing out the major effects that must be considered. Positive coefficients indicate that the corresponding experimental variables have a favourable effect on the system response, whereas the opposite trend is observed for the negative ones. Moreover, depending on the significance of each coefficient, which is determined via a statistical approach and related to the *p*-values, the influence of the investigated variables and their interactions can be easily evaluated in order to determine the main effects regulating the experimental response. In particular, a high coefficient significance (i.e., *p* < 0.001 corresponding to *** symbol) indicates that the related variable plays a fundamental role and should be held in great regard; contrariwise, the variable whose coefficients show a low significance (i.e., *p* > 0.05) do not considerably contribute to the system response and can be discarded in order to build a simplified mathematical model.

**Figure 6:**
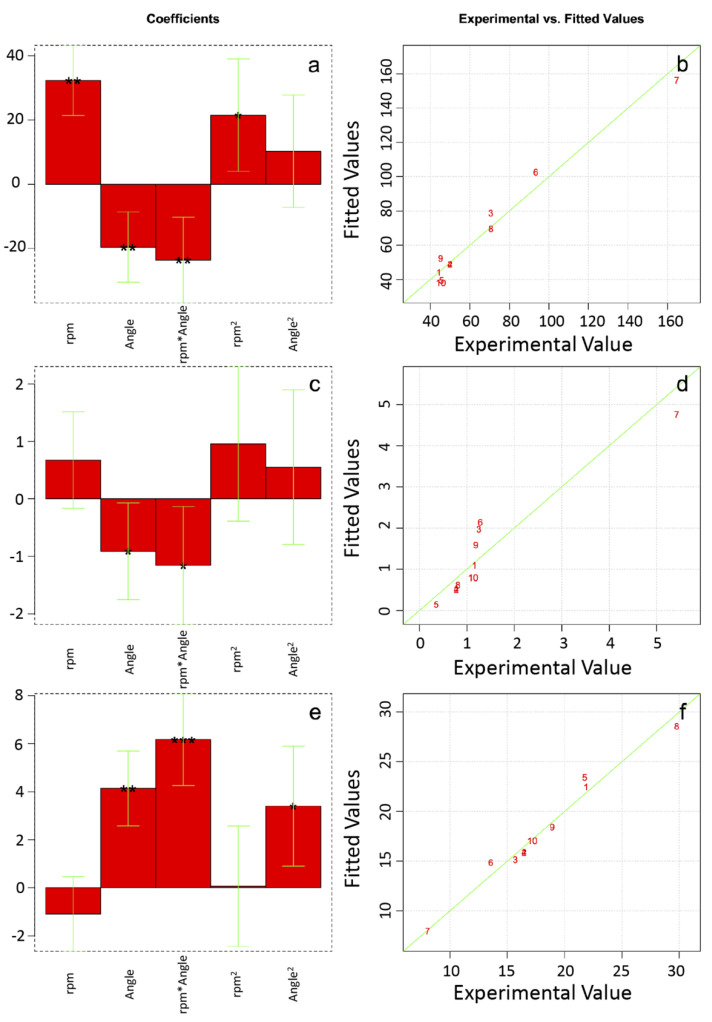
Model coefficients for the Young’s modulus (**a**), the maximum tensile strength (**c**), and the elongation at break (**e**); *** indicates a significance at *p* < 0.001, ** a significance at *p* < 0.01, and * a significance at *p* < 0.05. A comparison is shown between the experimental (numbers) and the theoretical (lines) values for the Young’s modulus (**b**), the maximum tensile strength (**d**), and the elongation at break (**f**).

The coefficients of the models derived from the experimental results are reported in Figure 6a,c,e for the Young’s modulus, the maximum tensile strength, and the elongation at break, respectively. 

Similar results were obtained, in terms of positive and negative coefficients for the Young’s modulus and the maximum tensile strength models. Nevertheless, a different significance can be observed. In particular, Y appears to be equally influenced by both the collector rotating speed (i.e., rpm), the direction of investigation (i.e., Angle), and their linear interactions (i.e. rpm*Angle), whereas the quadratic terms (i.e., rpm^2^ and Angle^2^) do not seem to play a very important role. Contrariwise, σ_r_ is mostly governed by the direction of investigation (i.e., Angle) and by the linear interaction between the variables, with the other terms being nearly negligible. The elongation at break is instead characterized by a different trend of the coefficients, with the linear interaction term (i.e., rpm*Angle) and the direction of investigation (i.e., Angle) being responsible for the main positive effects on the system response. 

To verify the consistency of the derived mathematical models, a comparison between the theoretical values and the experimental values is shown in Figure 6b,d,f for the Young’s modulus, the maximum tensile strength, and the elongation at break, respectively. Independently on the selected physical quantity, a good agreement was observed, thus proving the truthfulness of the obtained models and the potential of the presented approach.

**Figure 7:**
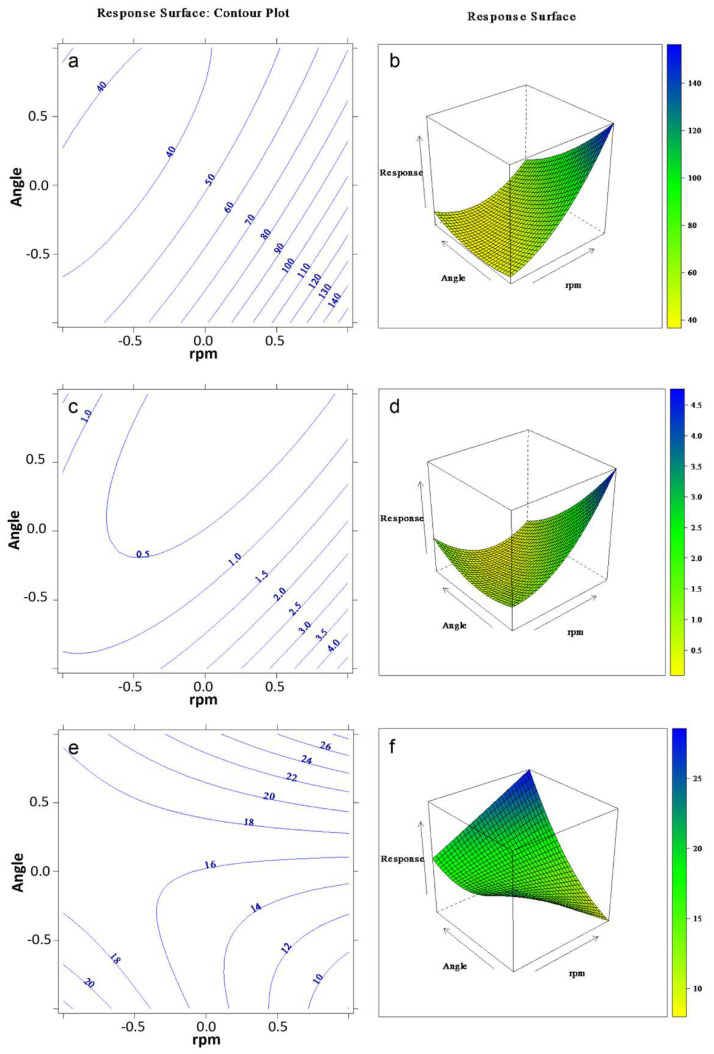
Response surfaces for the Young’s modulus (**a**,**b**), the maximum tensile strength (**c**,**d**), and the elongation at break (**e**,**f**).

An alternative to immediately and simply visualize the effects of the investigated variables without the need for a complex statistical analysis is represented by the contour plot sand the response surfaces, which are shown in Figure 7. 

Whereas the contour plots (Figure 7a,c,e) show the system response on two-dimensional graphs, the response surfaces (Figure 7b,d,f) provide an efficient tool to visualize the three-dimension response in the whole experimental domain, with the yellow and the blue colours indicating low and high values, respectively. As mentioned above, the Young’s modulus and the maximum tensile strength are characterized by a similar behaviour, with the response showing a more or less monomodal trend and increasing with the collector rotating speed (i.e., rpm) but decreasing with the direction of investigation (i.e., Angle). The elongation at break is instead characterized by a more complex behaviour, with the response surface showing a bimodal trend; in particular, the response appears to increase with decreasing the collector rotating speed (i.e., rpm) but increasing the direction of investigation (i.e., Angle), thus showing an opposite trend with respect to Y and σ_r_. Moreover, from the slopes and the curvatures of the represented surfaces, it can be easily noted that for the Young’s modulus and the maximum tensile strength, the main factor affecting the system response is related to the collector rotating speed (i.e., rpm); on the contrary, the interactions between the investigated variables (i.e., rpm*Angle) play the key role in conditioning the membrane deformation behaviour.

## 4. Conclusions

Polyvinylpyrrolidone electrospun membranes with different nanofiber orientations were prepared using a simple rotating drum collector setup. In particular, aligned nanofibers were obtained when increasing the rotating speed owing to the higher collecting surface tangential velocity; however, contrariwise to what expected, the alignment of the fibres was accompanied by an increase of the fibre diameter and a wider dimension distribution, which was most likely ascribable to an elastic shrinkage of the polymer chains within the nanofibers. The complete screening of the mechanical, dynamic-mechanical, and dynamic-mechanical-thermal properties was able to conclusively prove the strong effect of nanofiber organization on the sample behaviour. Indeed, besides higher Young’s modulus and maximum tensile strength values alongside the fibre alignment direction, improved mechanical properties were also observed transversely and perpendicularly due to the partially-oriented and more densely-packed polymer chains that provided a greater stiffness to the nanofibers themselves. In this regard, the dynamic-mechanical and the dynamic-mechanical thermal analysis evidenced good thermal stability of the prepared membranes and the presence of a secondary transition corresponding to a chain entropic relaxation and occurring at a lower temperature as their alignment increased. Finally, the applied design of experiment approach and the related mathematical models derived via statistical analysis successfully provided detailed information regarding the effects of the experimental variables and their linear interactions on the system response. In particular, whereas the Young’s modulus and the maximum tensile strength were highly related and showed the same behaviour being mostly determined by the collector rotating speed, the elongation at break was strongly influenced by the interactions between the two investigated variables. Such outcomes could not be easily extrapolated by the traditional data handling, thus highlighting the great advantages of this kind of approach, which, to the best of our knowledge, has not been employed before to deeply understand the mechanical behaviour of electrospun membranes with randomly- or uniaxially-oriented nanofibers. 

## Figures and Tables

**Table 1:** Summary of the investigated variables and their experimental ranges.

Factor	Variable	Unit	Low Level	High Level	Central Level
x_1_	Rotating speed	rpm	0	500	250
x_2_	Angle of investigation	degree (°)	0	90	45

**Table 2:** Summary of the mechanical properties of the prepared membranes in three different directions.

Rotating Speed (rpm)	Angle (°)	Y (MPa)	σ_r_ (MPa)	ε_r_ (%)
0	0 45 90	44 ± 1 45 ± 2 45 ± 2	1.2 ± 0.2 1.1 ± 0.2 1.2 ± 0.1	22 ± 5 17 ± 5 19 ± 4
250	0 45 90	71 ± 2 50 ± 1 46 ± 3	1.4 ± 0.1 0.8 ± 0.2 0.4 ± 0.1	16 ± 4 16 ± 5 22 ± 1
500	0 45 90	165 ± 6 93 ± 3 71 ± 3	4.0 ± 0.2 1.3 ± 0.1 0.8 ± 0.2	8 ± 1 14 ± 2 30 ± 2

**Table 3:** Summary of the dynamic-mechanical and dynamic-mechanical-thermal properties of the prepared membranes. E’ values are taken at a frequency of 1 Hz, and T_φ_ represents the secondary transition temperature.

Rotating Speed (rpm)	Angle (°)	E′ at T = −40 °C (MPa)	E′ at T = 20 °C (MPa)	E′ at T = 80 °C (MPa)	T_φ_ (°C)
**0**	0 90	48.3 ± 1.2 48.7 ± 0.9	42.4 ± 0.8 41.9 ± 0.2	39.5 ± 0.6 40.1 ± 0.4	45 ± 2 44 ± 3
250	0 90	79.5 ± 1.1 49.1 ± 1.4	69.6 ± 1.2 42.6 ± 1.5	62.7 ± 1.2 40.2 ± 3.0	24 ± 1 0 ± 3
500	0 90	186.1 ± 4.3 84.4 ± 3.6	160.4 ± 6.8 70.1 ± 4.3	142.4 ± 1.2 66.3 ± 1.1	5 ± 1 −10 ± 3
